# Supplementary material for: A pragmatic approach for producing theoretical syntheses in ecology
Source: PLoS One. 2021 Dec 17;16(12):e0261173. doi: 10.1371/journal.pone.0261173 (PMC8682896; doi:10.1371/journal.pone.0261173)
Supplement: S1 File — (PDF) [file pone.0261173.s001.pdf]

Supplementary File I – Application of the Nominal Group Technique

The application of the adapted NGT proceeded as follows:

1. Each volunteer of a trio register the propositions she/he identified as being the central contribution of the relevant publication to the collective of scientists under study based solely on her/his reading of the citations.
2. The individual propositions identified by each volunteer is presented to the entire trio, without each volunteer knowing the author of each proposition, except their own.
3. Each propositions is numbered.
4. All volunteers in the trio read the propositions of their peers in the trio.
5. Each volunteer in the trio reevaluates their proposition of what is the message central message of the citations based on their reading of the propositions identified her/his peers in the trio.
6. Each volunteer in the trio makes a new proposal of what the citations read conveyed as the central message of the focal relevant publication. In this proposal, the volunteers should inform which propositions should be combined into one or more complex mechanisms (here dubbed "models"), which should remain separated as a simple proposition and which should be discarded as redundant or irrelevant.
7. The proposals are then presented to the trio, again, without each volunteer knowing the author of each proposal, except by their own.
8. Each proposal is numbered.
9. Each volunteer reads the proposal of their peers in the trio.
10. The proposals are then ranked by each volunteer in a scale from the most adequate to least adequate according to what the set of citations read conveyed about the relevant publication.
11. The proposal with the higher rank considering the ranking of all three volunteers is selected.

12. If the winning proposal contains one or more models. The following steps ensue. If there is more than one model, the following steps ensue for each model. If there are no combination of propositions in the winning proposal, the simple propositions in the winning proposal are treated as final syntheses and we jumped directly to step 17.

13. Each volunteer produces an individual synthesis of the first model mentioned in the winning proposal of the step 11.

14. The syntheses made by each participant are presented to the trio, again, without each person knowing the author of each synthesis.

15. Each volunteer in the trio reads the syntheses of their peers in the trio.

16. The proposed syntheses are ranked and the synthesis with the higher rank is selected.

17. The winning synthesis is submitted to an open evaluation by the volunteers that propose modifications to it if they think necessary.

18. The modified synthesis are compared to the winning synthesis of the step 16 and re-ranked.

19. The process described in the steps 16 to 18 are repeated until consensus is reached.
