# Supplementary material for: A pragmatic approach for producing theoretical syntheses in ecology
Source: PLoS One. 2021 Dec 17;16(12):e0261173. doi: 10.1371/journal.pone.0261173 (PMC8682896; doi:10.1371/journal.pone.0261173)
Supplement: Attachment — Submitted filename: Response to reviewers.docx [file pone.0261173.s005.docx]

### Editor:

###### Comment E1:

The introduction is quite philosophical, and I wonder whether this is even necessary. When reading the title of your paper, readers will very likely expect a proposal for a new synthesis method. And ecological synthesis usually does not start with axioms or theories, but with data and empirical results of single studies, trying to bring them together to see the big picture. I recommend you to frame the introduction more towards clarifying how your method is situated in this context. Maybe the manuscript would even work well without any reference to the axiomatic approach?

**Reply:** We agree with that comment and substantially changed the introduction section. We removed the dichotomy between axiomatic and pragmatic views. In the current version, we focus mainly on why ecology could benefit from having a framework that is better aligned with its way of doing science. As a consequence, the introduction is much shorter and direct.

###### Comment E2:

Also, I was wondering if the method would be easier to understand if you would start with the case study. The chapter starting in line 72 is very abstract and I had problems figuring out how all this might work in practice. The brief summary of the relevant steps may work better in a chapter where you propose to go beyond the case study. There you could suggest to turn this into a method applicable to other cases as well. But this is just a suggestion - please try and see whether this works and is an improvement or not.

**Reply:** We understand that the description is somewhat abstract. We modified the text so the ideas in the description of the approach are less abstract. However, we decided not to change the order of the presentation of topics in the article because we worry that placing the case study first may convey the message that the specific method we used is what we are proposing as the approach.

Our main message is that the proposed approach can be implemented in different ways, and thus the specific method we used in our study case is just one way to do that. Understanding this difference has other implications too.

We think that in a situation in which a reader thinks we are trying to propose a specific method, reservations about how we chose to conduct this method might lead her/him to discard the PATh approach as a whole. However, there is two main advantages in conveying the message that the case study is simply a proposal of how the PATh can be implemented. First, if a reader has reservations about the specific method we used, this perspective invites readers to think of better ways to implement the approach we proposed. Additionally, if readers propose better ways to implement the PATh, these proposals give strength to the argument that the approach indeed facilitates the revision process of synthesis generation. Therefore, we decided to maintain the structure of the manuscript because we believe this is the best way to present the general approach and the method used in the case study separately.

Nevertheless we changed the text so that our intent to separate these things are clearer.

###### Comment E3:

As a side note, more related to the Oikos manuscript: your suggestions concerning a pragmatic, pluralist approach for ecology sound quite related to the following book, which you may not be aware of: Reiners, W. A., & Lockwood, J. A. (2010). Philosophical Foundations for the Practice of Ecology: Cambridge University Press.

**Reply:** After you mentioned it, I (Bruno) read this book and am very glad I did. Although the discussions about pluralism are not as complete as in Nancy Cartright’s work, I found that it is completely aligned with Lorraine Daston works about intersubjectivity, and more focused on the ecological perspective too. We decided to cite the book to make some of the arguments in the text more specific to ecology.

It is somewhat surprising that we have this kind of discussion in the literature for decades, and yet, most manuscripts in which “theories” are being proposed are about a specific type of model, namely, mathematical model (as shown by Marquet et al 2014).

###### Comment E4:

Reviewer #2 considers your method as too subjective and prone for biases. In my opinion, conceptual synthesis is something that needs to be done by researchers using their creative potential and their language interpretation skills, and statistics can only be helpful to some degree here. I agree with Reviewer #1 that the consensus approach you chose is a helpful way to counteract subjectivity in this process. In order to avoid arguments like those put forward by Reviewer #2, you may try to clarify early on that the aim here is not to synthesize data or reach consensus about evidence, but that the kind of synthesis your method is designed for is conceptual synthesis. Your method thus (as far as I understood) does not aim at competing with formal, statistical meta-analyses, but rather with narrative conceptual reviews (which are often done with what you call the expert approach).

**Reply:** Thank you for suggesting how to deal with the reviewer’s comment. As suggested, we added some sentences to make it clearer that the syntheses made by using the PATh are not meant to replace the approach that makes syntheses of data in section 2. However, we disagree that the lack of a statistical analysis is the same as the lack of a formal analysis and that a statistical analysis would make the synthesis less prone to personal biases. See the reply to Comment R2.1 and R2.2 in this document for more details of the argument.

###### Comment E5:

I very much agree with the comment by Reviewer #1 concerning generalizability of your approach. Please discuss this at some point in the manuscript. Ideally, you would come up with some suggestions for further applications of your method, to indicate whether it will always need to be such broad concepts, or whether you think this method could also work for synthesizing theory on more detailed research questions.

**Reply:** We agree that the generality of the approach needs to be presented more explicitly. We changed the last paragraph of the discussion to present the arguments we used to reply to Comment R1.2. Please, see the detailed response below.

###### Comment E6:

Also, please response to Reviewer #1’s comment concerning negative results and zombie ideas: Do you think these cases are covered during the step where you classified some of the studies as ‘negational’?

Reply: Thank you again for the suggestion of how to address the issue. The classification of citations into negational or supportive was to guarantee that the propositions identified were, in fact, being used to support the scientific activities of the focal collective. The problem referred to in this comment will only be an issue if people do not understand the kind of data the PATh generates. It is important to understand that the PATh will identify what is being used, regardless of the reasons for that. This implies that sometimes, it will identify models that are being used for reasons that are not aligned with our views of how science should be. Therefore, the results of the PATh cannot be used to validate knowledge as empirically sound (see reply to Comment R1.1). However, there is a vast discussion in the epistemology of science about whether empirical support should be considered the most important epistemological value in scientific research. There are other values in science, such as heuristics, aesthetics, and simplicity. The PATh can be used to show that such models are in fact being used, and how they are being used. If one thinks a model is being used for the wrong reasons, we believe the PATh can help to spot and describe this more precisely, and thus will show what needs to be changed, according to that view.

In the current version of the manuscript, this aspect of this synthesis is discussed thoroughly in the 6th paragraph of the discussion section.

### Reviewer 1

###### Comment R1.1:

This approach is rather descriptive because is based on quantifying propositions “relevance” via number of citations (which is fully acknowledged by the authors), but how do you deal with “popular” but “outdated or wrong” propositions that have been perpetuated in current literature? For example, there was a discussion on ecology about the usefulness of the “intermediate disturbance hypothesis” stating that larger diversity is expected at intermediate levels of disturbance. The point of that discussion is that this hypothesis has been disproved several times, however, due to its simple and powerful prediction, it is still cited. Similar cases have been named “zombie ideas” that are still cited but they should no longer be. My point is, could this method run the risk of legitimizing propositions that are proven false on close examination, but people still cite and use them?

**Reply:** We completely understand this concern, and in fact we think our approach can contribute to spot propositions that are problematic, according to any view. Therefore, we do not think this will be a problem, provided people understand what the PATh is supposed to achieve. It is supposed to show us what models a determined community is using to learn about phenomena, independently of the reasons this community is using them. Because a community can make use of models for different reasons (which might be wrong reasons for a given view of science or theory), the information of which models are in use does not validate these models as empirically sound. However, this information can be used to propose changes and discuss future developments in the field.

For instance, the reviewer argued that the “intermediate disturbance hypothesis” should not be used to build new models because empirical evidence does not support it. Nevertheless, the PATh has shown that this model is frequently used, which may help to make the point on “zombie ideas”, if you wish. We think that the method can be useful to highlight the role of models for a given community, and thus allow us to scrutinize causes and consequences of such roles. We agree that the idea of “zombie ideas” is one among many possibilities, and we are glad to know that the reviewer had proposed this link. .

Comment R1.2:

is this method only useful for “defining” concepts. Your example is about how do you define “succession”. I think this is very useful, I can think of tons of concepts in ecology that could benefit from it (like “symbiosis”), however how could the method be used for more specific cases? For example, what about doing a theoretical synthesis on the “conditions that stabilize mutualisms”? or “the emergence of cheaters in symbiotic relationships in ecosystems”? Most commonly, reviews in ecology address such kind of questions, rather than a definition of a concept… do you think your method could also be extended to those cases? It would be great to have some lines on the generalizability of your approach in the discussion.

Reply: We are very grateful for the reviewer for thinking of new ways in which our approach could be useful. We believe these examples could be addressed by using the PATh. We changed the text to make this possibility more clear in the text and also included the lines the reviewer suggested and, in fact, used one of the examples proposed by the reviewer.

The main objective of the approach is to identify what models are being actually used to give support to studies conducted by a specified community of scientists. It is perfectly feasible to define keywords to survey the studies about “conditions to stabilize mutualisms”. After that, the steps of the PATh could be followed in the exact same way we conducted in our study to reach a set of models in use in this field. In the present study, we found it interesting to discuss the definition of the phenomenon by the focal collective, the dichotomy between what is in textbooks and what is being actually used, and how the neutral model is permeating a field that is historically based on niche. However, it is not warranted that the set of models used as conceptual basis in other fields of study will lead to the same observation we made.

We’ve already applied the PATh to make a synthesis of the “species coexistence” domain. The definition of keywords involved a little more debate because there is no single word to define the phenomenon (at least not an obvious one like “succession”). We had to identify some common concepts around this phenomenon and then think of keywords to survey the studies about the phenomenon. After that, we followed the approach in the exact same way, which led us to a set of models in use. This set of models in use, as expected, is revealing facts about the theory in use about species coexistence that are different from what the models of the current manuscript revealed about the succession domain. We are currently writing the manuscript about this implementation of the PATh

Comment R1.3:

I really think the abstract does not give merit to the paper. It is written in such a general way, that is unclear how unique is the approach. For example, I could not find the key argument that “proposition relevance” is measured by how “frequently those propositions are referred”.

Reply: We are thankful to the reviewer for making us aware of that. We changed the abstract so it is more descriptive of the Approach. This includes the information that the frequently referred publications are the ones used to survey the conceptual bases of the field of study.

Comment R1.4:

I have problems with wording like “information and knowledge” or “what is being used by this collective”. It would be better to use “propositions” directly.

**Reply:** Agreed. In the previous version, we decided not to use simply “propositions” in some places because propositions are a statement that can be true or false, which is one of the ways in which knowledge transits among studies. However, we are essentially identifying propositions. Therefore, we believe that changing the manuscript as suggested greatly improved textual clarity without much loss of conceptual clarity.

Comment R1.5:

I think lines in the introduction (L47-50) and L60-66 would be great in the abstract. I think those lines summarize that a) ecology is pragmatic and not axiomatic, meaning that the most common propositions used by ecologist reflects a decision among these scientists on what verbal models are useful in order to understand a phenomenon; b) thus, a key challenge is to identify and quantify those propositions; c) your contribution is that you develop a method to achieve exactly that identification and quantification.

**Reply:** We appreciate this suggestion. The new version of the abstract tries to convey that information more clearly.

Comment R1.6:

L4: I suggest stating as sentence that the paragraph is about showing examples of how challenging is to make synthesis about theory in ecology. The reason for my suggestion is that after reading the sentence “However, the conceptual bases of knowledge generation in ecology might not be easy to identify” I was expecting an explanation of why is that the case. Instead, what is mentioned is a list of failed attempts to reach such identification.

L12. Related to my previous comment, I suggest here the first sentence should state that here is where the reason for this problem (the identification of the conceptual bases of ecology) is explained. Sth like: “Part of the problem in making theoretical synthesis in ecology is that scientists force this field to be axiomatic. We argue that this field is pragmatic.”

**Reply:** Agreed. Following the suggestion of the editor, we changed the text to make it more accessible to our readership. We removed the paragraph explaining in details the differences between axiomatic and pragmatic views, giving emphasis to the task of synthesizing ecological theory and how it can be made. We believe that this version has more internal coherence within paragraphs.

Comment R1.7:

L70. Here you mention that you will discuss how your approach is different from “other approaches”. I thought that was really cool, but after reading the paper I realize you actually only compare to one specific case (the one used by Pickett, Meiners and Cadenasso). Unless I missed more approaches discussed, I think you should state within your case study, you compare your approach to another study that have similar aims but used a distinct approach.

**Reply:** That is exactly the message we would like to convey, and we thank the reviewer for letting us know that it was not clear enough.. We changed the text to “ These results are used to discuss in what way our proposed approach to producing a theoretical synthesis is different from another study with the same aim but that used the more traditional “expert opinion” approach. We end up presenting how syntheses produced by using our approach can fulfil the role of a clearly enunciated theory.”

Comment R1.8

L85. This is a typo; it should say “definition of the collective of agents”.

**Reply:** This typo was corrected.

### Reviewer 2

Comment R2.1: I appreciate the efforts of the authors for proposing a more methodological way to gather and summarise knowledge in ecology. I enjoyed reading about their example on succession and I found the findings reasonable based on my knowledge of the field.

My main concern is about the reproducibility of the findings. As the authors say, the synthesis produced will strongly depend on the collective of scientists that has been identified (step 2) as well as by the group of people used to synthesize the information (step 6). Unless multiple comparisons are made among different groups/regions/etc, the syntheses produced could be highly biased.

**Reply:** We understand this concern as it was one of ours through the development of the approach and we agree that the previous version of the manuscript could be confusing in the methodological descriptions of the steps that dealt with these issues. We thank the reviewer for calling attention to these points. We significantly changed the section describing the content analysis of citations to convey more clearly why and how we conducted each of these steps.

The dependence of the results of the approach on the collective of scientists and on the group of people used to synthesize the information are of different natures. The pragmatic view of theories assumes that scientists decide what to use to learn about phenomena, therefore, a different set of scientists might use a different set of models to learn about a phenomenon. This implies that different collectives of scientists may have different theories about a phenomenon. This is not a bias of the approach, this is a property of how theories develop according to the pragmatic view.

Multiple comparisons among different groups would indeed be very informative about particularities of how a determined phenomenon is studied across social groups. However, these multiple comparisons are not necessary to understand the views of the defined collective of scientists. The aim of the PATh is to identify what a determined community of scientists uses as conceptual bases to learn a phenomenon. Defining what community one intends to analyse is not assuming a bias it is defining the object of the study.

The group analysis step (6) is intended to control for known individual biases. Every theoretical synthesis has biases , and our approach is one of the few approaches to produce theoretical syntheses that acknowledges that and has a methodological step to make it less biased.

The confidence in the final models is grounded on how we process actual data using an intersubjective approach (see Daston 1992 cited in the manuscript for a discussion about subjectivity in science). The citations are actual data coming from written documents that can be inspected. A group of people reads the same citations and makes individual syntheses based on this reading. The individual syntheses are as biased as any interpretation of text found throughout the literature. These individual syntheses are then subjected to a consensus analysis where they are compared for similarities and differences. After scrutinizing what was individual interpretation and real content coming from the citations, new collective syntheses are proposed. This reduces the individual biases of interpretation of the citations (see Bernard & Ryan, 2009, for more details on the assumptions of the method).

Comment R2.2: The lack of statistical or any kind of formal analyses at any step of the process increases this concern.

**Reply:** We did not conduct any quantitative analysis, but we did conduct a series of qualitative analysis to ground our results on actual data. It is important to note that our synthesis is not aimed at replacing data syntheses, such as meta-analysis. Rather, it is supposed to be complementary to such syntheses. However, it is important to remember that statistical analyses cannot give objectivity to a study. Statistical analysis uses a series of conventions to reach a consensus about quantities necessary to interpret data (see Daston 1992). We used a formal analysis to reach a qualitative consensus about data, this analysis allows us to have confidence in our interpretation of the data used, because it is standardized and reproducible, and also addresses sources of subjectivity.

We also added some sentences regarding that matter in the section describing the general approach.

Comment R2.3: The approach they propose could be used to allow a more direct and consistent comparison among reviews and syntheses of the field, but I am unsure of the generality of the syntheses produced as they would be strongly dependent on personal views since there is no objective or statistical way to analyse the data collected.

**Reply:** We completely understand this concern, but we disagree that our approach is strongly dependent on personal views, for the reasons we detailed in the previous comment. As with any other approach to make conceptual syntheses, it still counts with human expertise and intuition. However, different from other approaches, ours is not only based on that. Another advantage of it is that the confidence in the result is not based on the renown of the ones proposing it, but on the method in itself. There are different ways to execute each step, therefore, if one is not confident in the results, she/he can point out which step of the method has a problem.
