# Supplementary material for: A pragmatic approach for producing theoretical syntheses in ecology
Source: PLoS One. 2021 Dec 17;16(12):e0261173. doi: 10.1371/journal.pone.0261173 (PMC8682896; doi:10.1371/journal.pone.0261173)
Supplement: Attachment — Submitted filename: PATh.docx [file pone.0261173.s006.docx]

I have enjoyed reading this MS despite holding the belief that such frameworks of theories may indeed, as the authors point out, sometimes restrain knowledge and development of a field such as ecology.

It is however clear that the authors have endeavoured to satisfy previous reviewer comments, notably on the clarity of the manuscript, which is now appropriate for publication in my opinion. I do not find the current MS too philosophical, the language used is apt, and should be understood and appreciated by a wide audience. Moreover, the figures well represent the MS and add clarity. I would therefore recommend this MS for publication, though I do have a few suggestions that ought to be addressed first.

I agree with previous reviewers that the abstract still does not add merit to the MS. In particular, the two final sentences are too vague. It is important to state here what PATh enables/improves specifically that was previously not possible/clear.

I agree with the editors comment that the method is somewhat prone to bias, please add a sentence to the discussion to clarify if the outcome might have differed using a different makeup (age, level of qualification/position, interest) of researchers.

Some small changes to be made at the authors discretion as follows:

1. Consider changing ‘produce’ to ‘producing’ in the title.

2. Line 52 and elsewhere ‘the field’ to most ecologists means surveying/sampling. Please specify ‘this field’ or ‘ecology’

3. Line 53 consider changing ‘scientific activity in the area’ to ecological activity/science’

4. Line 78 make clear that this general approach can be applied to specific topics/sub-fields within ecology.

5. Line 175 ‘path’ should be ‘PATh’.

6. Line 226 ‘created a cut-off’ should be ‘used/adopted a cut-off’

7. Line 275 ‘Thus, we adopted’

8. Line 580 ‘Hacking’ reference should be reformatted.

9. Throughout the article, ‘Fig’ should be ‘Fig.’

10. Supplementary 1, Point 3. ‘Each proposition’
