# Supplementary material for: A pragmatic approach for producing theoretical syntheses in ecology
Source: PLoS One. 2021 Dec 17;16(12):e0261173. doi: 10.1371/journal.pone.0261173 (PMC8682896; doi:10.1371/journal.pone.0261173)
Supplement: Attachment — Submitted filename: Response to reviewers_3.docx [file pone.0261173.s007.docx]

### Editor:

###### Comment E1:

The paper has improved evidently and the contents are worth of interest for the community. There are however still points to be addressed before the manuscript can reach an acceptable standard for being published.

The material in the paper looks interesting, but the manuscript need to be further revised carefully to meet PLOS ONE publication criteria.

Please take particular care to the comments raised by Reviewer 4.

In particular make sure to have data available, you should present the "real data" used on your synthesis.

**Reply:** All data used in the analysis is described in the manuscript or in the supplementary material. We believe that the perception that the whole data was not available was caused by a mislabelling of supplementary material, which has been corrected. Please see a more thorough explanation in the replies to Comment R4.2.

In terms of the results of the synthesis, the paper could benefit from more context and explanation, proving that your final results give evidence that your synthesis was the best approach in your analysis.

**Reply:** We understand that most manuscripts describing methodological approaches are aimed at proving that the described approach is in fact the best when compared to other approaches. However, what makes this approach more adequate to deal with the theoretical synthesis in ecology is the fact that it is based on the pragmatic view of theories. As we described in the introduction, it is difficult to produce a theoretical synthesis of a field that develops pragmatically. Nevertheless, ecology develops pragmatically as we argue in Travassos-Britto et al, 2021. Our approach is the only one, to our knowledge, that is aligned with the pragmatic view of theories in ecology. Therefore, the objective of the manuscript is to argue that it is possible to implement such an approach, to give examples of the kind of information we can generate with it, and to describe how different this approach is from other approaches because of the pragmatic assumption. In other words, our results from the case study are not intended to prove that this is the best approach, they are intended to exemplify how we can use the approach to further knowledge in this field.

The structure of the manuscript was thought to convey this message. In the introduction, we set the problem with syntheses based on axiomatic views of theories, in the “PATh – Pragmatic Approach to Theories” we describe the general premises behind the approach; In the “A case study on ecological succession” we describe the way we choose to implement it; in the “Pragmatic theory of succession” we describe the types of insights we can have by using the method and in the “Discussion” section we present how different this approach is from approaches based on the axiomatic premises.

We modified the new version of the manuscript to make it clear. In the new version, the beginning of each of these sections makes it clear that this is the objective of this manuscript.

### Reviewer 3

###### Comment R3.1:

I have enjoyed reading this MS despite holding the belief that such frameworks of theories may indeed, as the authors point out, sometimes restrain knowledge and development of a field such as ecology.

**Reply:** We understand the view that theories restrain knowledge development because theory is used as a guide and this may make it more difficult to see what is not described in the guide. However, we believe that syntheses produced by this approach are definitely less restraining than syntheses proposed by other known approaches. First, because PATh syntheses are explicitly descriptive. In this sense, they state that this is what people are doing, not necessarily how the user of the synthesis should do. Second, the synthesis is based on the premise that we don’t need theories unified by axioms to produce knowledge. Therefore, in a way, this kind of synthesis encourages scientists to make decisions based on what they think it’s useful to learn about the natural world. Third, if theories may restrain knowledge development in some sense, we also cannot truly progress in producing knowledge without the conceptual and methodological focus provided by theories. Thus, it is not so much about regretting that theories guide our views on phenomena, but about finding the proper balance between the focused view theories allow us and the shifts that transformations of our perspectives on phenomena can give us.

###### Comment R3.2:

It is however clear that the authors have endeavoured to satisfy previous reviewer comments, notably on the clarity of the manuscript, which is now appropriate for publication in my opinion. I do not find the current MS too philosophical, the language used is apt, and should be understood and appreciated by a wide audience. Moreover, the figures well represent the MS and add clarity. I would therefore recommend this MS for publication, though I do have a few suggestions that ought to be addressed first.

I agree with previous reviewers that the abstract still does not add merit to the MS. In particular, the two final sentences are too vague. It is important to state here what PATh enables/improves specifically that was previously not possible/clear.

**Reply:** We appreciate that the reviewer acknowledged our effort towards reaching a wider audience, as we detailed in our comments to the Editor. We made modifications in the abstract to be more precise in what differentiates this approach from other ways to produce syntheses.

###### Comment R3.3:

I agree with the editors comment that the method is somewhat prone to bias, please add a sentence to the discussion to clarify if the outcome might have differed using a different makeup (age, level of qualification/position, interest) of researchers.

**Reply:** We are not sure if the reviewer refers to a bias caused by the makeup of the community of scientists under study or whether it refers to a bias caused by the makeup of the volunteers involved in producing the syntheses. If it’s the latter, we agree that the outcome may vary with the makeup of the synthesis group. We think this is an expected feature of our approach, which is conceived to allow groups to build their own syntheses on how theory of a given field is (or was) structured. Of course, in many cases it is to share such syntheses to a wider audience, but as the approach provides all data (e.g. references, excerpts) the resulting synthesis is open to scrutiny. The item 1 of the nominal group technique description states about the risk of this bias (see lines 300-312 of the Main MS)

If the bias is referring to the makeup of the scientific community under study. I think the reply to the Comment R2.1 of the previous round addresses this issue in a very thorough manner. I pasted it below for easier consultation.

*[[Comment R2.1: I appreciate the efforts of the authors for proposing a more methodological way to gather and summarise knowledge in ecology. I enjoyed reading about their example on succession and I found the findings reasonable based on my knowledge of the field.*

*My main concern is about the reproducibility of the findings. As the authors say, the synthesis produced will strongly depend on the collective of scientists that has been identified (step 2) as well as by the group of people used to synthesize the information (step 6). Unless multiple comparisons are made among different groups/regions/etc, the syntheses produced could be highly biased.*

***Reply:*** *We understand this concern as it was one of ours through the development of the approach and we agree that the previous version of the manuscript could be confusing in the methodological descriptions of the steps that dealt with these issues. We thank the reviewer for calling attention to these points. We significantly changed the section describing the content analysis of citations to convey more clearly why and how we conducted each of these steps.*

*The dependence of the results of the approach on the collective of scientists and on the group of people used to synthesize the information are of different natures. The pragmatic view of theories assumes that scientists decide what to use to learn about phenomena, therefore, a different set of scientists might use a different set of models to learn about a phenomenon. This implies that different collectives of scientists may have different theories about a phenomenon. This is not a bias of the approach, this is a property of how theories develop according to the pragmatic view.*

*Multiple comparisons among different groups would indeed be very informative about particularities of how a determined phenomenon is studied across social groups. However, these multiple comparisons are not necessary to understand the views of the defined collective of scientists. The aim of the PATh is to identify what a determined community of scientists uses as conceptual bases to learn a phenomenon. Defining what community one intends to analyse is not assuming a bias it is defining the object of the study.*

*The group analysis step (6) is intended to control for known individual biases. Every theoretical synthesis has biases , and our approach is one of the few approaches to produce theoretical syntheses that acknowledges that and has a methodological step to make it less biased.*

*The confidence in the final models is grounded on how we process actual data using an intersubjective approach (see Daston 1992 cited in the manuscript for a discussion about subjectivity in science). The citations are actual data coming from written documents that can be inspected. A group of people reads the same citations and makes individual syntheses based on this reading. The individual syntheses are as biased as any interpretation of text found throughout the literature. These individual syntheses are then subjected to a consensus analysis where they are compared for similarities and differences. After scrutinizing what was individual interpretation and real content coming from the citations, new collective syntheses are proposed. This reduces the individual biases of interpretation of the citations (see Bernard & Ryan, 2009, for more details on the assumptions of the method). ]]*

###### Comment R3.4:

Some small changes to be made at the authors discretion as follows:

1. Consider changing ‘produce’ to ‘producing’ in the title.

2. Line 52 and elsewhere ‘the field’ to most ecologists means surveying/sampling. Please specify ‘this field’ or ‘ecology’

3. Line 53 consider changing ‘scientific activity in the area’ to ecological activity/science’

4. Line 78 make clear that this general approach can be applied to specific topics/sub-fields within ecology.

5. Line 175 ‘path’ should be ‘PATh’.

6. Line 226 ‘created a cut-off’ should be ‘used/adopted a cut-off’

7. Line 275 ‘Thus, we adopted’

8. Line 580 ‘Hacking’ reference should be reformatted.

9. Throughout the article, ‘Fig’ should be ‘Fig.’

10. Supplementary 1, Point 3. ‘Each proposition’

**Reply:** All suggestions were accepted. We are thankful to this reviewer for the careful reading of our manuscript.

### Reviewer 4

I was excited by the idea of this paper. I feel that this could be a unifying way to synthesize the literature in ecology. However, I felt that the paper had several shortcomings:

###### Comment R4.1

1)Need to better set up the theory in ecological succession before using it as the example. Why ecological succession chosen for this study? Need more background here.

**Reply:** Agreed. In the section “Defining the phenomenon of interest”, in which we explain why we chose ecological succession as the object of study, we added an explanation regarding the domain history. In fact, one of the reasons why we chose this phenomenon was its long history and its numerous theoretical syntheses proposed in the past. We changed the manuscript to make such reasoning clearer.

###### Comment R4.2

2) Need to provide the results. Is there a table of the results of your analyses.

**Reply:** All data used in the study are available either in the manuscript or as supplementary material. We identified that the previous versions of the manuscript had some mislabelling of supplementary material. We apologize for this issue, which is now corrected.

Regardless, we think it is worth clarifying that the manuscript describes a new approach thought to be aligned with the pragmatic view of theories and, therefore, it might not follow any previously used standard description of methods. We will take this opportunity to respond to each question individually explaining where we made results available and why.

Comment R4.2.1:How many paper fell into each category by evaluators?

**Reply:** The papers were not divided into categories. Each relevant publication had a set of citing publications that could vary in size. The following description was conducted by each of the relevant publications. First, we identified the set of publications of the scientific community that cited the focal relevant publication. Then, in these publications, we identified phrases or paragraphs in which the focal relevant publication was cited and excerpted these snippets of text referring to the relevant publication. For each relevant publication, there were 50 of these snippets excerpted from the publications published by the focal community. To produce an individual synthesis of the model in use originated in a relevant publication, a volunteer read these 50 excerpts of text. All excerpts of text read by the volunteers are described in Supplementary Material 3 labelled by the relevant publication they refer to. This process is completely described in detail in the section “Analyzing the content of citations” in topics (lines 300-341 of the Main MS).

Comment R4.2.2:How were the citations distributed?

**Reply:** Citations were distributed according to what is described in Table 1 of the MS (line 285). It is important to note that these are not general citations of each relevant publication. These are the citations made by the focal scientific community under study. We changed the caption of the table to make it more clear.

Comment R4.2.3: What were the most common propositions of relevant publications?

**Reply:** The most relevant propositions of each relevant publication are described in the textual models. A textual model can be composed of a single proposition or a set of propositions that work together as a nomological machine. We describe this concept in detail in the reply to Comment R4.3.2, we also added a more clear definition in the MS (342-348).

Comment R.4.2.4: What were common themes in textual syntheses?

**Reply:** Although we agree that a thematic analysis could reveal interesting things about the similarities among textual models, we did not believe that such an analysis would be necessary to reach our goal. We used a consensus analysis based on an adaptation of a nominal group technique. The exact method we used is described in the Supplementary material 1.

Comment R4.2.5: What were the 29 textual models?

**Reply:** Each of the 29 models is described in supplementary material 2. We agree that the main result of the approach is the set of 29 textual models and it might be unorthodox to list it in the supplementary material. However, each of the 29 models is at least one paragraph long. Therefore, adding it to the main text could make it too long for a standard paper of PLOS One. We included this information in the main manuscript (Lines 369-371), so it is clear to readers that the main results are in the Supplementary Material.

###### Comment R4.3

Comment R4.3.1: 3) You in text example could be taken out of paper, Lines 341-353. I did not follow what you synthesized from the text and how you identified the 29 verbal models?

**Reply:** I believe some lack of clarity is justified in the fact that there was some mislabeling of the supplementary material in the MS. This is actually one example of the 29 models reached. This textual description of a mechanism was the result of following steps 1 to 6 of the approach. This text was synthesized by the volunteers based only on their reading of the excerpts of text referring to Connel & Slatyer (1977). It is important to note that the evaluators did not know to which publication the excerpts of text were referring to when they read it and some evaluators have had none or almost no contact with this literature. Therefore, this verbal model is the actual result of a synthesis based only on the reading of citing excerpts of texts referring to Connel & Slatyer (1977). It is not (and should not be) based on the previous knowledge of what Connel and Slatyer wrote in their paper. The process to generate such model is described in detail in the section “Analyzing the content of citations”

We used this model in the MS to exemplify what are the verbal models resulting from this process, the rest of the models are described in supplementary material 2. We think the clearer definition of the verbal models (lines 343-346) and the clearer explanation that the other models are in the Supplementary Material should make this clear.

Comment R4.3.2: I don't follow some of your terminology. Verbal models? textual models? how do they differ? These need to be better described.

**Reply:** We agree that there were no clear definitions for these terms. We were using them as synonyms. We changed the MS to use just “verbal models” and gave a proper definition in lines 336-340. Thank you for alerting us.

###### Comment R4.4

4) Your "Propositions" (Lines 380-392): I found these to be confusing. Why are they not written in terms of the ecological theory in ecology. The way that they are phrased does not sound like ecology.

**Reply**: We understand that these propositions are not written as one might find in classical literature in ecology. The phrasing is a result of the method described in the first paragraph of the section “Defining ecological succession”.We reassessed the method and the sentences to check if we could change it to a more familiar phrasing. We found the phrasing of these propositions the most direct and unequivocal way to make the statements without contradicting any of the 29 models identified.

###### Comment R4.5

What is your final synthesis? What models are best? I need the "so what" for ecology. State the important gains from your study.

**Reply:** We think that the reviewer was precluded from understanding all our results due to the mislabeling of the supplementary material. We corrected this issue and we think that these questions are properly addressed in the paper.

In short, the objective of the approach is not to indicate the best models to learn about a phenomenon. It is rather to map how a specific community of scientists is using models to learn about the phenomenon. The resulting verbal models synthesize the views of the selected community about ecological succession. These models reveal major tendencies of model use within the scientific community, as we discussed in the section “Pragmatic theory of succession”. This, in no way, is a recommendation of the best models to understand succession (as described in the second to last paragraph of the Discussion section). However, understanding these tendencies gives us a clear picture of the state of research within a domain of study, which should help us better guide the future of the domain.

###### Comment R4.6:

Any meta-analyses?

**Reply:** We do think that a meta-analysis could be an interesting way to contrast what we’ve found with quantitative data. The meta-analysis and the PATh, however, have very distinct objectives. While the former is an approach that assesses confidence in statistical analysis about a hypothesis, the latter is an approach to assess how models and their subjacent propositions are being used as conceptual bases to learn about a phenomenon. At the moment, we are studying how we can combine the two approaches to check if quantitative data gives support to what is being used by the scientific community. I think the reply to Comment R2.2 from previous rounds of review can give a clear insight into how we see it.

Comment R2.2: The lack of statistical or any kind of formal analyses at any step of the process increases this concern.

**Reply:** We did not conduct any quantitative analysis, but we did conduct a series of qualitative analysis to ground our results on actual data. It is important to note that our synthesis is not aimed at replacing data syntheses, such as meta-analysis. Rather, it is supposed to be complementary to such syntheses. However, it is important to remember that statistical analyses cannot give objectivity to a study. Statistical analysis uses a series of conventions to reach a consensus about quantities necessary to interpret data (see Daston 1992). We used a formal analysis to reach a qualitative consensus about data, this analysis allows us to have confidence in our interpretation of the data used, because it is standardized and reproducible, and also addresses sources of subjectivity.

We also added some sentences regarding that matter in the section describing the general approach.
